# Assessing pyrethroid resistance in *Aedes aegypti* from Cordoba Colombia: Implications of *kdr* mutations

**DOI:** 10.1371/journal.pone.0309201

**Published:** 2024-08-22

**Authors:** María Claudia Atencia–Pineda, Diana Diaz-Ortiz, Paula Pareja–Loaiza, Javier García–Leal, Richard Hoyos–López, Alfonso Calderón–Rangel, Pedro Fragozo-Castilla, Lisandro Pacheco-Lugo, Adriana E. Flores, Ronald Maestre–Serrano

**Affiliations:** 1 Doctorado en Microbiología y Salud Tropical, Facultad de Medicina Veterinaria y Zootecnia, Universidad de Córdoba, Montería, Colombia; 2 Facultad de Ciencias Básicas y Biomédicas, Centro de Investigación en Ciencias de la Vida (CICV), Universidad Simón Bolívar, Barranquilla, Colombia; 3 Facultad de Ciencias de la Salud, Centro de Investigación en Ciencias de la Vida (CICV), Universidad Simón Bolívar, Barranquilla, Colombia; 4 Instituto de Investigaciones Biológicas del Trópico (IIBT), Universidad de Córdoba, Montería, Colombia; 5 Grupo de Investigación Parasitología Agroecología Milenio, Universidad Popular del Cesar, Valledupar, Colombia; 6 Facultad de Ciencias Biológicas, Universidad Autónoma de Nuevo León, San Nicolás de los Garzas, México; University of Ibadan Faculty of Science, NIGERIA

## Abstract

Resistance to insecticides is one of the great challenges that vector control programs must face. The constant use of pyrethroid-type insecticides worldwide has caused selection pressure in populations of the *Aedes aegypti* vector, which has promoted the emergence of resistant populations. The resistance mechanism to pyrethroid insecticides most studied to date is target-site mutations that desensitize the voltage-gated sodium channel (VGSC) of the insect to the action of pyrethroids. In the present study, susceptibility to the pyrethroid insecticides permethrin, lambda-cyhalothrin, and deltamethrin was evaluated in fourteen populations from the department of Córdoba, Colombia. The CDC bottle bioassay and WHO tube methods were used. Additionally, the frequencies of the F1534C, V1016I, and V410L mutations were determined, and the association of resistance with the tri-locus haplotypes was examined. The results varied between the two techniques used, with resistance to permethrin observed in thirteen of the fourteen populations, resistance to lambda-cyhalothrin in two populations, and susceptibility to deltamethrin in all the populations under study with the CDC method. In contrast, the WHO method showed resistance to the three insecticides evaluated in all populations. The frequencies of the mutated alleles ranged from 0.05–0.43 for 1016I, 0.94–1.0 for 1534C, and 0.01–0.59 for 410L. The triple homozygous mutant CIL haplotype was associated with resistance to all three pyrethroids evaluated with the WHO bioassay, while with the CDC bioassay, it was only associated with resistance to permethrin. This study highlights the importance of implementing systematic monitoring of kdr mutations, allowing resistance management strategies to be dynamically adjusted to achieve effective control of *Aedes aegypti*.

## Introduction

*Aedes aegypti* is the vector of the main arboviruses of greatest importance in public health, such as dengue, Zika, chikungunya, and yellow fever. However, it has also been found that this species is infected with the Eastern equine encephalitis virus, Venezuelan equine encephalitis virus, Rift Valley fever virus, La Crosse virus, Potosí virus, and Oropouche virus, among others [1]. Vector competition studies have shown that it can transmit the Mayaro virus [2] and the West Nile virus [3].

The ecological plasticity of *Ae*. *aegypti*, the resistance of eggs to desiccation, urbanization, and changes in climatic conditions have allowed it to colonize new urban and rural environments, expanding its distribution and colonization areas worldwide. Currently, it is distributed in 114 countries in tropical and subtropical regions. This wide distribution has caused an increase in cases of dengue, Zika, and chikungunya throughout the world [4].

In 2022, the Americas reported 2,803,096 cases of dengue, 36,370 of Zika, and 271,006 of chikungunya. During this period, Colombia stood out as the country with the second-highest number of cases of severe dengue in the Americas, with 1,371 cases, only surpassed by Brazil. Likewise, Colombia reported the simultaneous circulation of the four serotypes of the dengue virus [5]. In 2023, Colombia reported 131,784 cases of dengue, representing a national incidence of 368.6 cases per 100,000 inhabitants, a substantial increase in incidence compared to 2022, when it was 177.5 cases per 100,000 inhabitants [6].

Among the aforementioned national cases of dengue, 4,191 were reported in the department of Córdoba, where 49.7% were classified as cases without warning signs, 49.4% with warning signs, and 0.8% corresponded to severe cases of dengue, with an incidence of 224.3 cases per 100,000 inhabitants [7]. This represents an increase compared to 2022, where 3,876 cases were reported, with an incidence of 208.8 cases per 100,000 inhabitants [6]. Regarding cases of chikungunya and Zika, from the 2014 and 2015 epidemics until 2022, approximately 16,882 cases of chikungunya and 4,108 of Zika were recorded. After the epidemic phase of these two diseases, annual cases have remained below 50, according to the epidemiological surveillance system of the National Institute of Health of Colombia (SIVIGILA).

To date, vector control has been established as the main strategy to reduce vector populations and interrupt the transmission cycle during epidemics of these arboviruses. This prevalence is due to the absence of specific treatments and effective vaccines for these diseases, especially in vulnerable populations [8]. The strategies adopted to reduce vector populations include the elimination of breeding sites, environmental management, community education, biological control, and the use of insecticides [9]. Recently, innovative techniques have been explored, such as the release of sterile male insects-SIT, insects carrying the lethal gene RIDL, and insects infected with Wolbachia, among others [10].

In Colombia, vector control has integrated the use of organochlorine, carbamate, organophosphate, and pyrethroid insecticides. However, in the 1990s, pyrethroids stood out in vector control due to their effectiveness against insects, low toxicity for mammals, and rapid environmental degradation. Their application through ultra-low volume fumigations, impregnated tarps, and household aerosols [8, 11] has exerted selection pressure on *Ae*. *aegypti* populations, generating the development of populations resistant to these insecticides in different regions of the country [12], representing a challenge for vector control programs. For several years, the organophosphates malathion, pirimiphos-methyl, temephos, and, to a lesser extent, the pyrethroids lambda-cyhalothrin and deltamethrin have been used in the country for vector control because of reports of resistance [8], along with the biological insecticide *Bacillus thuringiensis* var. *israeliensis* and growth regulators such as pyriproxyfen and diflubenzuron [13].

Insecticide resistance is mediated by various mechanisms, including physiological and/or behavioral changes in insects, reduced penetration or absorption through the cuticle, increased metabolic detoxification mediated by enzymes such as cytochrome P450 (CyP450), esterases, and glutathione-S-transferase, and point mutations at the active site, which results in lower sensitivity to insecticides [14, 15]. The most studied mechanisms of resistance to pyrethroid insecticides include mutations in the voltage-gated sodium channel (VGSC) and alteration in the levels of enzymes that detoxify xenobiotics [14, 16].

The Knockdown resistance (*kdr*) mutations F1534C, V1016I, and V410L have been reported in populations of *Ae*. *aegypti* from Colombia, to be associated with pyrethroid resistance [9, 12, 17–21]. In addition, glutathione-S-transferases, alpha-esterases, and CyP450 oxidases have been identified in the vector populations [9, 12, 17, 22].

In Córdoba, research focused on evaluating the susceptibility of vector populations to insecticides has been limited. Only two studies have reported on resistance to organophosphate and pyrethroid insecticides, in addition to *kdr* mutations and alterations in detoxification enzymes, specifically in the municipality of Montería [12, 17]. Additionally, data from the insecticide resistance surveillance network in Colombia reveal resistance to fenitrothion in San Bernardo del Viento and to pirimiphos-methyl in Pueblo Nuevo. However, these findings do not reflect the current susceptibility to pyrethroids or the resistance mechanisms in most of the municipalities of Córdoba. This information is crucial for vector control programs in the department since it would contribute significantly to developing and strengthening public policies for controlling dengue, Zika, and chikungunya.

This study aimed to evaluate the current state of susceptibility to permethrin, deltamethrin, and lambda-cyhalothrin and identify the presence of the F1534C, V1016I, and V410L mutations in VGSC. It also investigated their possible relationship with resistance in populations of *Ae*. *aegypti* from the department of Córdoba, Colombia.

## Materials and methods

### Study area

**Fig 1:**
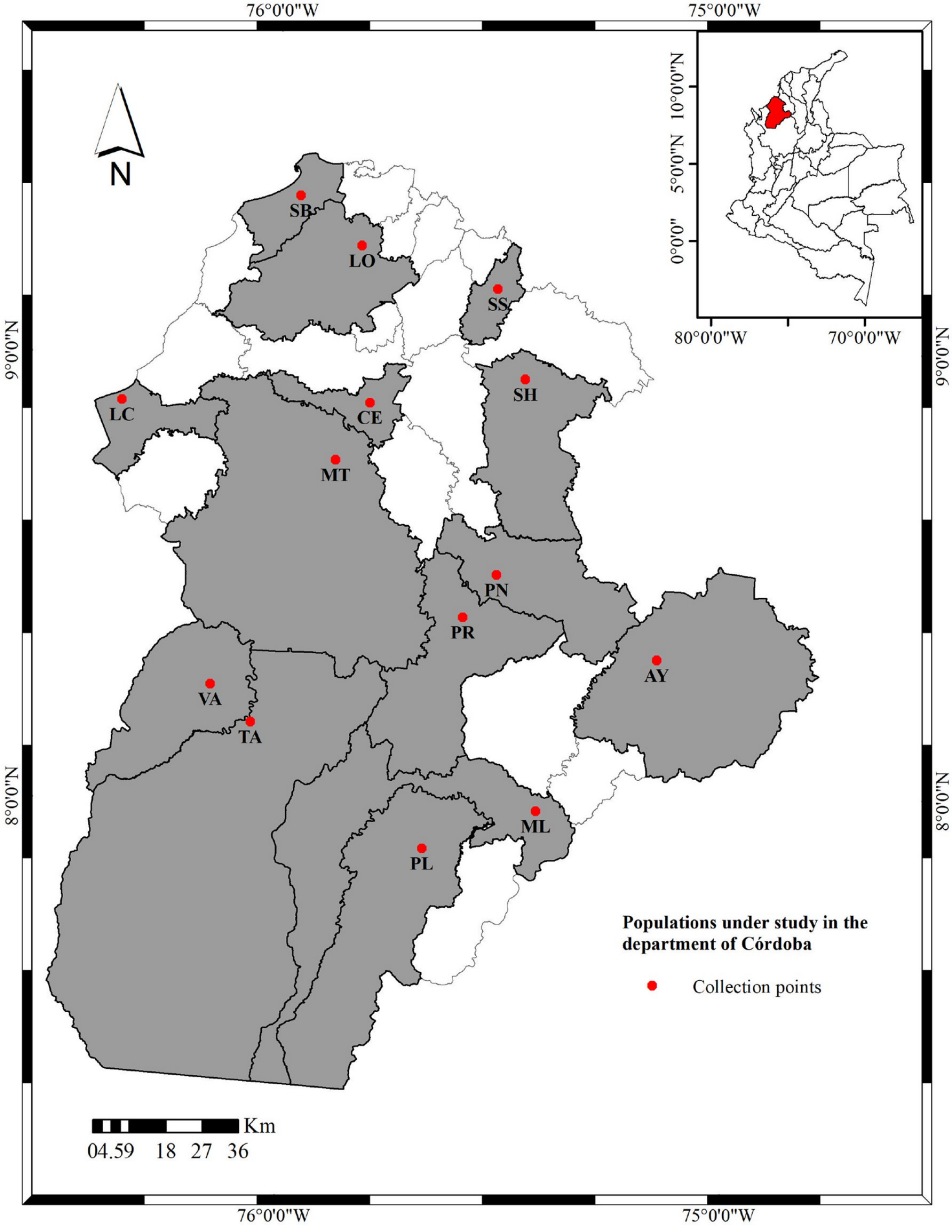
Map of the department of Córdoba showing the study populations. CE: Cereté, SH: Sahagún, PR: Planeta Rica, SB: San Bernardo del Viento, LO: Lorica, AY: Ayapel, ML: Montelíbano, TA: Tierralta, PN: Pueblo Nuevo, VA: Valencia, MT: Montería, SS: San Andrés de Sotavento, LC: Los Córdobas, PL: Puerto Libertador. Source: taken from Instituto Geográfico Agustín Codazzi (IGAC). CC BY 4.0 License https://www.colombiaenmapas.gov.co/?e=-82.43784778320864,-0.17644239911865092,-71.23179309571162,9.90326984502256,4686&b=igac&u=0&t=23&servicio=204.

The study was carried out in the department of Córdoba during 2022 and 2023; this department is situated in the northwestern part of Colombia on the Caribbean Plain (132,000 km^2^) at 7°22’05”N to 9°26’16”N and 74°47’43”W to 76°30’01”W, and it covers an area of 23,980 km^2^. Mosquito populations of *Ae*. *aegypti* were collected in the municipalities of Montelíbano (7°58′16″N, 75°25′5″W), Sahagún (8°57′2″N, 75°26′44″W), Lorica (9°14′19″N, 75°48′50″W), San Bernardo del Viento (9°21′18″N, 75°57′16″W), Tierralta (8°10′22″N, 76°3′34″W), Pueblo Nuevo (8°30′18″N, 75°30′27″W), Planeta Rica (8°24′32″N, 75°34′55″W), Cereté (8°53′12″N, 75°47′28″W), Valencia (8°15′33″N 76°08′49″ W), Ayapel (8°18′45″N, 75°8′42″W), Puerto Libertador (7°53′17″N, 75°40′18″W), San Andrés de Sotavento (9°08′43″N, 75°30′31″W), Los Córdobas (8°53′43″N, 76°21′17″W) and Montería (8°45′36″N, 75°53′8″W) (Fig 1), where there is the presence of the vector and circulation of dengue, chikungunya, and Zika viruses.

### Mosquito collection and obtaining F_1_/F_2_

To collect the immature forms of *Ae*. *aegypti*, entomological inspections were carried out in homes in selected neighborhoods in fourteen municipalities of Córdoba. Verbal informed consent was obtained from the residents before collecting mosquitoes outside their homes, ensuring their approval and understanding of the study’s purpose and procedures. All possible containers, including pools, cans, tires, and bottles, were inspected. Entomological material was collected in 300 to 400 sites distributed in the urban area. The larvae and pupae were transported in containers of approximately 5 L to the insectaries of the Simón Bolívar Universities and Universities of Córdoba. The larvae were fed with dog food and the adults with an artificial feeder (methodology endorsed by the ethics committee of Simon Bolivar University with code CEI-USB-CE-0369-00-00). Where the F1 and F2 generations were obtained under controlled conditions of temperature (28 ± 2°C), relative humidity (60 ± 10%), and 12 h:12 h light: dark photoperiod.

### CDC and WHO bioassays

The bioassays were carried out using the F1 and F2 generations of *Ae*. *aegypti* from the 14 selected populations. Using the CDC method [23], the doses and diagnostic time of the insecticides permethrin (15 μg/bottle; 30 min), deltamethrin (10 μg/bottle; 30 min), and lambda-cyhalothrin (10 μg/bottle; 30 min) were evaluated, which were prepared from technical grade insecticides (ChemService, West Chester, PA, USA). A bottle with acetone, without insecticide, was used as a control bottle. In those populations where resistance was detected, the intensity of resistance was determined in the same way by exposing mosquitoes to two (2×), five (5×), and ten (10×) times the diagnostic dose [24].

Furthermore, using the WHO-impregnated paper tube method [24], the discriminating concentrations of permethrin (0.40%), deltamethrin (0.03%), and lambda-cyhalothrin (0.05%) were evaluated, recording the results after one hour of exposure and again 24 h post-exposure. Paper without insecticides was used as a control. Live and dead individuals from both the CDC and WHO bioassays were phenotypically categorized as resistant (R) or susceptible (S) and were stored individually at -80ºC in 0.5 ml tubes for genotypic analysis of the *kdr* mutations V1016I, F1534C, and V410L.

All the procedures described above were performed in all field populations of *Ae*. *aegypti* and the susceptible Rockefeller strain as a reference.

The interpretation of the results obtained through the CDC and WHO bioassays was established as follows: mortalities between 98–100% indicated susceptibility, between 90–97% suggested possible resistance that must be confirmed, and mortalities <90% were interpreted as resistance (S1 File) [24].

In cases where the mortality observed in the control group exceeded 10%, the bioassay was invalidated. On the other hand, mortalities in the control between 5 and 10% were corrected for the observed mortality using the Abbott formula [24].

### Detection of *kdr* alleles

Using the quantitative polymerase chain reaction (qPCR) technique, the V1016I, F1534C, and V410L mutations in VGSC were identified, and the allelic and genotypic frequencies were calculated. Accordingly, forty F0 parental mosquitoes of *Ae*. *aegypti* of each study population were selected. Likewise, these same mutations were identified in all mosquitoes, which, according to the CDC and WHO methods, were classified as phenotypically resistant (R) and susceptible (S) to the insecticides evaluated.

To identify the mutations, each mosquito was processed in duplicate, and in each test, three positive controls—a wild homozygote, dominant homozygote, and heterozygote—were included, in addition to a negative control consisting of a mixture without DNA template.

DNA was extracted from *Ae*. *aegypti* using the Quanta Biosciences Extracta^™^ Kit, following the manufacturer’s instructions. The DNA obtained was quantified in a Nanodrop ND-ONE-W spectrophotometer from Thermo Scientific.

PCR reactions were carried out using a CFX96 Real-Time System C1000 thermal cycler from Bio-Rad. To determine the genotypes of loci 1016, 1534 and 410, dissociation curve analyses were used. The specific amplification of the V1016I mutation, located in exon 21 of VGSC gene, was performed following the protocol established by Saavedra-Rodríguez et al. [25]. The total volume of the reaction mixture was 20 μL, composed of 9.2 μL of deionized water (ddH2O), 10 μL of iQ_TM_ SYBR1 Green Supermix (Bio-Rad), 0.1 μL of each of the specific primers V1016f, I1016f and I1016r at a concentration of 50 μM and 0.5 μL of DNA (Table 1). The amplification program consisted of an initial denaturation step at 95°C for 3 min, followed by 40 cycles of 95°C for 10 s, 60°C for 10 s, and 72°C for 30 s, and a final extension at 72°C for 10 min. The melting curves would be determined using a denaturation gradient from 65 to 95°C with increments of 0.2°C every 10 s.

**Table 1:** Primer sequences used for genotyping *kdr* mutations.

Mutation	Primer	Sequence (5´ – 3´)
**V1016I**	V1016(f)	5´-CGGGCAGGGCGGCGGGGGCGGGGCCACAAATTGTTTCCCACCCGCACCGG-3´
	I1016(f)	5´-GCGGGCACAATTGTTTCCCACCCGCACTGA-3´
	I1016(r)	5´-GGATGAACCGAAATTGGACAAAAGC-3´
**F1534C**	C1534(f)	5´-GCGGGCAGGGCGGCG GGGGCGGGGCCTCTACTTTGTGTTCTTCATCATGTG-3´
	F1534(f)	5´-GCGGGCTCTACTTTGTGTTCTTCATCATATT-3´
	F1534(r)	5´-TCTGCTCGTTGAAGTTGTCGAT-3´
**V410L**	V410(f)	5´- GCGGGCAGGGCGGCGGGGGCGGGGCCATCTTCTTGGGTTCGTTCTACCGTG-3´
	L410(f)	5´- GCGGGCATCTTCTTGGGTTCGTTCTACCATT-3´
	L410(r)	5′-TTCTTCCTCGGCGGCCTCTT-3′

To detect the F1534C mutation, the method described by Yanola et al. [26] was followed and adapted to the conditions of our laboratory. A final reaction volume of 20 μL was used, composed of 6 μL of ddH2O, 9 μL of iQ_TM_ SYBR1Green Supermix (Bio-Rad), 0.8 μL of primer C1534f, and 0.6 μL of primers F1534f and F1534r at a concentration of 10 μM, and 3 μL of DNA (Table 1). The amplification protocol began with an initial denaturation phase at 95°C for 3 min, followed by 37 cycles of 95°C for 10 s, 57°C for 30 s, and 72°C for 30 s, and a final extension at 72°C for 4 min. Melting curves were determined using a denaturation gradient from 65 to 95°C with an increase of 0.5°C every 5 s.

The V410L mutation was detected as described by Haddi et al. [27]. The final reaction volume was 21 μL, composed of 9.6 μL of ddH2O, 10 μL of iQ_TM_ SYBR1 Green Supermix (Bio-Rad), 0.1 μL of each of the primers L410f, and V410f, and 0.2 μL of the primer L410r at a concentration of 50 μM and 1.0 μL of DNA (Table 1). The amplification procedure began with an initial denaturation step at 95°C for 3 min, followed by 39 cycles of 95°C for 10 s, 60°C for 10 s, and 72°C for 30 s, and a final extension at 72°C for 30 s. The melting curves were determined using a denaturation gradient from 65 to 95°C with an increase of 0.2°C every 10 s.

The interpretation of the calibration curves was carried out with the Bio-Rad Precision Melt Analysis software as follows: for the V1016I mutation, a peak at 79 °C corresponds to a homozygous mutant (I/I), at 86 °C to a wild-type homozygote (V/V) and at 79 and 86 °C to a heterozygote (V/I); for the F1534C mutation, a peak at 84 °C corresponds to a mutant homozygote (C/C), at 81 °C to a wild-type homozygote (F/F) and at 84 and 81 °C to a heterozygote (F/C); and for the V410L mutation, a peak at 83 °C corresponds to a mutant homozygote (L/L), at 86 °C to a wild-type homozygote (V/V) and at 83 and 86 °C to a heterozygote (V/L).

From the parental F0 mosquitoes, the allelic frequencies of I1016, C1534 and L410 and genotypic frequencies for V_1016_/V_1016_, F_1534_/F_1534_, V_410_/V_410_, I_1016_/I_1016_, C_1534_/C_1534_, L_410_/L_410_, V_1016_/I_1016_, F_1534_/C_1534_ and V_410_/L_410_ were determined in each population studied (S1 File). The inbreeding coefficient was also estimated, thus providing a measure of internal genetic variation and possible self-consanguinity within the analyzed populations.

### Association analysis using odds ratio (OR)

Odds ratio (OR) analysis was used to investigate the relationship between haplotypes and resistance to the insecticides lambda-cyhalothrin, deltamethrin, and permethrin in the study populations. This method allows us to quantify the association between the presence of a specific haplotype and the survival of individuals (indicative of resistance to the above insecticides) compared to mortality (indicative of sensitivity to these insecticides).

The population (the total populations) was segmented into groups according to the identified haplotypes. Within each haplotype, mosquitoes that survived (resistant) and those that died (susceptible) were recorded (S1 File). The association between resistance phenotypes (WHO and CDC bioassay) and haplotypes (kdr frequencies) was assessed according to the odds ratio (OR), and statistical significance was determined using the Fisher exact probability test (α = 0.05).

### Ethics statement

This study has the endorsement of the Ethics Committee of Simon Bolivar University with code CEI-USB-CE-0369-00-00.

## Results

Using the CDC method (1), we evaluated a total of 3,255 females of *Ae*. *aegypti* against the insecticides permethrin (n = 1,106), deltamethrin (n = 1,069) and lambda-cyhalothrin (n = 1,080). In the case of permethrin, mortality percentages indicative of resistance were obtained in several populations: Montería (15.8%), Sahagún (16.8%), Cereté (20.2%), Ayapel (49.8%) and Valencia (64.2%) followed by Montelíbano, San Bernardo del Viento, Los Córdobas, Planeta Rica and Pueblo Nuevo with mortalities between 84.6 and 88.2%. Furthermore, in Lorica (94.9%), Puerto Libertador (92.3%), and Tierralta (92.2%), mortality percentages were observed that suggested possible resistance. Regarding deltamethrin and lambda-cyhalothrin, all the populations evaluated were susceptible, except Cereté (90.3%) and Montería (92.4%), which showed possible resistance to lambda-cyhalothrin (Table 2).

**Table 2:** Susceptibility of *Aedes aegypti* females from different locations in Córdoba, Colombia, to the diagnostic doses of the insecticides permethrin, deltamethrin, and lambda-cyhalothrin using the CDC method (1).

Populations	Pyrethroid					
	Permethrin		Deltamethrin		Lambda-cyhalothrin	
	1X DD		1X DD		1X DD	
	DD: 15 ug/bottle;		DD: 10 ug/ bottle; DT: 30 min		DD: 10 ug/ bottle;	
					DT: 30 min	
	DT: 30 min					
	n	%	n	%	n	%
Cereté	74	20.2	78	100	62	90.3
Ayapel	79	49.8	82	100	81	100
Sahagún	74	16.8	79	100	76	98.6
Planeta Rica	80	87.7	78	100	80	100
Pueblo Nuevo	82	88.2	66	100	75	100
Valencia	77	64.2	77	100	80	100
Tierralta	76	92.2	75	100	76	100
Montelíbano	85	84.6	83	100	86	100
Lorica	79	94.9	73	100	76	98.7
San Bernardo del Viento	82	85.3	83	100	75	100
Montería	77	15.8	68	98.5	79	92.4
Los Córdobas	82	86.2	74	100	76	100
San Andrés de Sotavento	83	98.8	72	100	79	100
Puerto Libertador	76	92.3	81	100	79	100

n: Number of mosquitos evaluated; %: Mortality percentage; DD: Diagnostic doses; DT: Diagnostic time.

In the populations identified as resistant to permethrin and lambda-cyhalothrin, the intensity of resistance was evaluated. For permethrin, 100% mortality was recorded in Tierraalta, Lorica, and Los Córdobas populations at twice the diagnostic dose (2x). Similarly, 100% mortality was recorded in the populations of Ayapel, Valencia, Montelíbano, San Bernardo del Viento, and Puerto Libertador at five times the diagnostic dose (5x). By increasing the diagnostic dose to ten times (10x), a mortality of 97.5% was observed in the population of Cerete, 98.7% for Sahagún, and 90.7% for Montería. For lambda-cyhalothrin, the populations of Cereté and Montería recorded 100% mortality when exposed to double the diagnostic dose (2x) (Table 3).

**Table 3:** Intensity of resistance of *Aedes aegypti* females from different locations in Córdoba, Colombia, to the insecticides permethrin and lambda-cyhalothrin using the CDC method (1).

Populations	Pyrethroid							
	Permethrin						Lambda-cyhalothrin	
	2X DD		5X DD		10X DD		2X DD	
	DD: 30 ug/bottle;		DD: 75 ug/bottle;		DD: 150 ug/bottle; DT: 30 min		DD: 20 ug/bottle;	
	DT: 30 min		DT: 30 min				DT: 30 min	
	n	%	n	%	n	%	n	%
Cerete	69	54.7	77	87.1	78	97.5	81	100
Ayapel	70	92.9	75	100	-	-	-	-
Sahagún	79	89.8	79	95.9	75	98.7	-	-
Planeta Rica	83	93.7	70	98.6	-	-	-	-
Pueblo Nuevo	81	90.3	71	98.6	-	-	-	-
Valencia	84	97.7	78	100	-	-	-	-
Tierralta	76	100	-	-	-	-	-	-
Montelíbano	71	91.6	76	100	-	-	-	-
Lorica	76	100	-	-	-	-	-	-
San Bernardo del Viento	78	91.1	76	100	-	-	-	-
Montería	77	77.9	67	80.1	67	90.7	73	100
Los Córdobas	81	100	-	-	-	-	-	-
San Andrés de Sotavento	-	-	-	-	-	-	-	-
Puerto Libertador	90	97.9	78	100	-	-	-	-

n: Number of mosquitos evaluated; %: Mortality percentage; DD: Diagnostic doses; DT: Diagnostic time.

**Fig 2:**
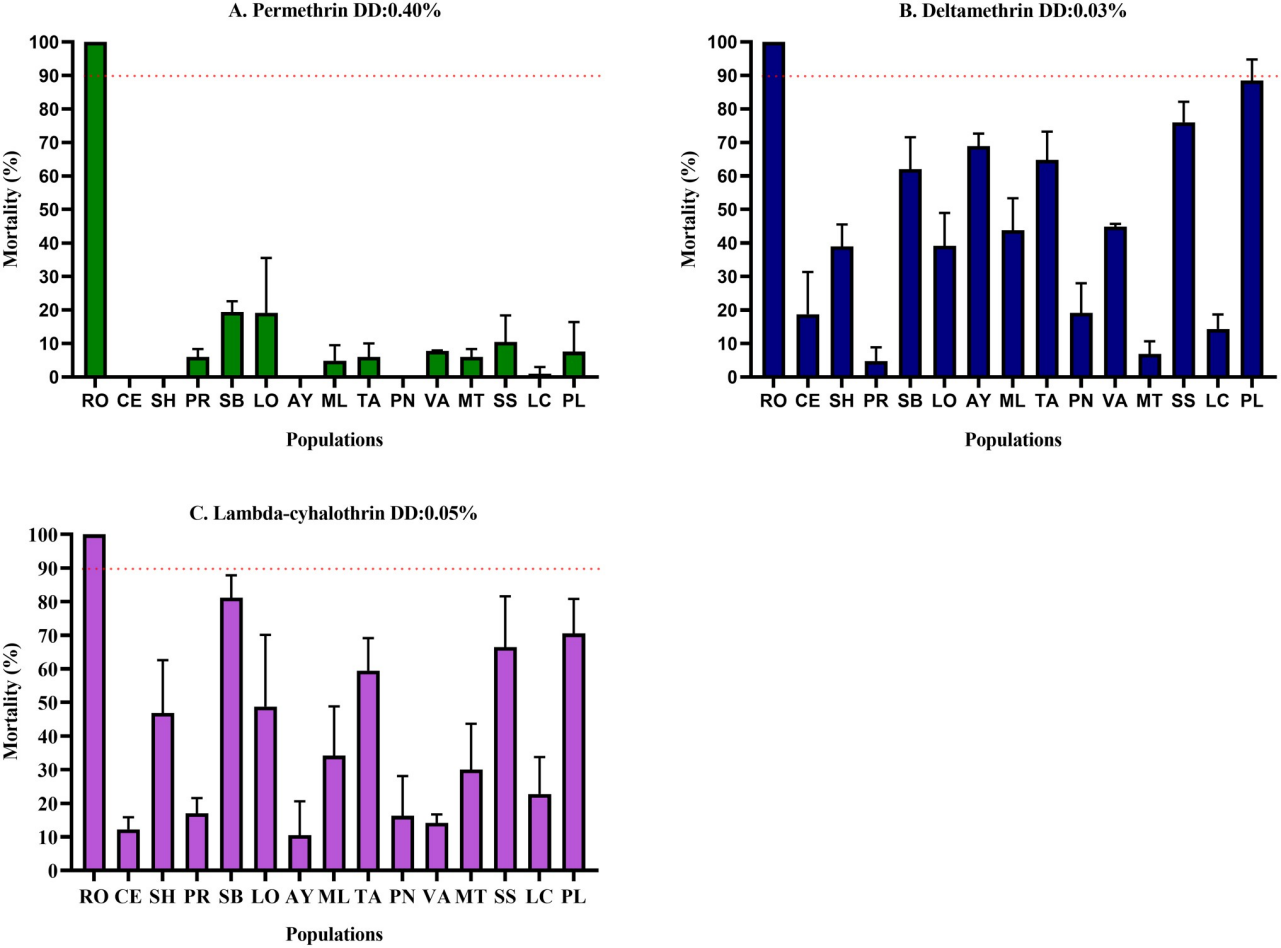
Susceptibility of *Aedes aegypti* females from different locations in Córdoba, Colombia, to the discriminating concentrations of permethrin (A), deltamethrin (B), and lambda-cyhalothrin (C) using the WHO technique (2). The mortality percentage was recorded 24 hours post-exposure. Interpretation criteria recommended by the WHO: susceptibility: mortality between 98–100%. Possible resistance that needs confirmation is mortality between 90–97%. Resistance: mortality <90%. RO: Rockefeller, SB: San Bernardo del Viento, LO: Lorica, PL: Puerto Libertador, CE: Cereté, ML: Montelíbano, TA: Tierralta, PR: Planeta Rica, PN: Pueblo Nuevo, SH: Sahagún, SS: San Andrés de Sotavento, AY: Ayapel, LC: Los Córdobas, VA: Valencia, MT: Montería.

Using the WHO standardized bioassay, we evaluated the effect of 4,030 females of *Ae*. *aegypti* to the insecticides permethrin (n = 1,342), deltamethrin (n = 1,368), lambda-cyhalothrin (n = 1,320) (S1 Table). Resistance to all three insecticides was confirmed in all populations examined. In the case of permethrin, the populations of Cereté, Ayapel, Sahagún, and Pueblo Nuevo recorded mortality percentages of 0%, while the other populations displayed a mortality ranging between 1 and 19.42%. For deltamethrin, the populations of Planeta Rica and Montería showed mortalities of 4.7 and 6.8%, respectively. The other populations evaluated exhibited low mortality percentages that varied between 14.3 and 76%, except the population of Puerto Libertador, where a rate of 88.4% was recorded. In the case of lambda-cyhalothrin, the populations of Ayapel and Cereté recorded mortality percentages of 10.5 and 12.2%, respectively. The other populations recorded mortalities between 16.2 and 70.5%, highlighting San Bernardo del Viento, with a mortality of 81.1% (Fig 2).

### Allelic and genotypic frequencies of *kdr* mutations V1016I, F1534C, and V410L

Mutations V1016I, F1534C, and V410L were identified in all *Ae*. *aegypti* populations from Córdoba. For the V1016I mutation, the three genotypes (VV_1016_, VI_1016_, and II1016) were detected in the populations of Cereté, San Bernardo del Viento, Valencia, Montería, Los Córdobas, and Lorica. The prevalence of the mutant genotype II_1016_ in the population of Montería stood out, with a frequency of 0.35, followed by Cereté and San Bernardo del Viento with frequencies of 0.20 and 0.10, respectively. In the other populations, this genotype was found to have a low frequency or was absent. The I_1016_ mutant allele showed the highest frequency in Montería 0.60, followed by Cereté and San Bernardo del Viento, with 0.43 and 0.40, respectively.

For the F1534C mutation, the three genotypes (FF1534, FC1534, and CC1534) were identified in all populations, although they did not co-occur. The mutant genotype CC_1534_ and allele C_1534_ showed high frequencies, between 0.88 and 0.98, in the populations of Valencia, Cerete, San Andrés de Sotavento, Montelíbano, and Planeta Rica. These were fixed (frequencies of 1.0) in Sahagún, San Bernardo del Viento, Tierralta, Puerto Libertador, Montería, Lorica, and Pueblo Nuevo populations.

Regarding the V410L mutation, genotypes VV_410_, VL_410_, and LL410 were detected in Valencia, Los Córdobas, Lorica, San Bernardo del Viento, Cereté, and Montería. The mutant genotype LL_410_ predominated in Montería, Cereté, and San Bernardo del Viento populations, with frequencies of 0.32, 0.18, and 0.10, respectively. The mutant allele L_410_ mainly occurred in Montería, Cereté, and San Bernardo del Viento populations, with frequencies of 0.59, 0.40 and 0.39, respectively.

When analyzing the inbreeding coefficients for the I1016 and L410 mutations, negative values were recorded, indicating an excess of heterozygotes in most populations, except Cereté, Los Córdobas, and Lorica, where the values were positive, indicating a heterozygous deficiency for both mutations. In the case of the F1534C mutation, the homozygous recessive genotype CC_1534_ was found fixed in the populations of Sahagún, San Bernardo del Viento, Tierralta, Puerto Libertador, Montería, Lorica and Pueblo Nuevo. On the other hand, in the populations of Los Córdobas, Montelíbano, San Andrés de Sotavento, and Valencia, negative values were observed, indicating excess heterozygotes (Table 4).

**Table 4:** Genotypic and allelic frequencies for the V1016I, F1534C, and V410L mutations in the *Aedes aegypti* populations of the department of Córdoba, Colombia.

**Populations**	**N**	**Genotype frequency**			**Allele frequency**		**F**_**IS**_
		**V1016I**			**V1016I**		
		**VV**	**VI**	**II**	**V**	**I**	
Sahagún	40	0.82	0.18	0.00	0.91	0.09	-0.096
Ayapel	40	0.78	0.22	0.00	0.89	0.11	-0.10
Cereté	40	0.35	0.45	0.20	0.57	0.43	0.07
San Bernardo del Viento	40	0.30	0.60	0.10	0.60	0.40	-0.25
Tierralta	40	0.72	0.28	0.00	0.86	0.14	-0.16
Puerto Libertador	40	0.48	0.52	0.00	0.74	0.26	-0.35
San Andrés de Sotavento	40	0.97	0.03	0.00	0.99	0.01	-0.01
Valencia	40	0.63	0.35	0.02	0.80	0.20	-0.09
Montería	40	0.15	0.50	0.35	0.40	0.60	-0.04
Los Córdobas	40	0.82	0.15	0.03	0.91	0.09	0.21
Montelíbano	40	0.85	0.15	0.00	0.93	0.07	-0.08
Lorica	40	0.65	0.30	0.05	0.80	0.20	0.06
Planeta Rica	40	0.90	0.10	0.00	0.95	0.05	-0.05
Pueblo Nuevo	40	0.65	0.35	0.00	0.82	0.18	-0.21
		**F1534C**			**F1534C**		**F**_**IS**_
		**FF**	**FC**	**CC**	**F**	**C**	
Sahagún	40	0.00	0.00	1.00	0.00	1.00	-
Ayapel	40	0.05	0.00	0.95	0.05	0.95	1.00
Cereté	40	0.02	0.00	0.98	0.02	0.98	1.00
San Bernardo del Viento	40	0.00	0.00	1.00	0.00	1.00	-
Tierralta	40	0.00	0.00	1.00	0.00	1.00	-
Puerto Libertador	40	0.00	0.00	1.00	0.00	1.00	-
San Andrés de Sotavento	40	0.00	0.02	0.98	0.01	0.99	-0.01
Valencia	40	0.00	0.12	0.88	0.06	0.94	-0.07
Montería	40	0.00	0.00	1.00	0.00	1.00	-
Los Córdobas	40	0.00	0.07	0.93	0.04	0.96	-0.04
Montelíbano	40	0.00	0.02	0.98	0.01	0.99	-0.01
Lorica	40	0.00	0.00	1.00	0.00	1.00	-
Planeta Rica	40	0.02	0.00	0.98	0.02	0.98	1.00
Pueblo Nuevo	40	0.00	0.00	1.00	0.00	1.00	-
		**V410L**			**V410L**		**F**_**IS**_
		**VV**	**VL**	**LL**	**V**	**L**	
Sahagún	40	0.82	0.18	0.00	0.91	0.09	-0.09
Ayapel	40	0.80	0.20	0.00	0.90	0.10	-0.11
Cereté	40	0.37	0.45	0.18	0.60	0.40	0.06
San Bernardo del Viento	40	0.32	0.58	0.10	0.61	0.39	-0.21
Tierralta	40	0.75	0.25	0.00	0.87	0.13	-0.14
Puerto Libertador	40	0.48	0.52	0.00	0.74	0.26	-0.36
San Andrés de Sotavento	40	0.97	0.03	0.00	0.99	0.01	-0.01
Valencia	40	0.62	0.35	0.03	0.80	0.20	-0.09
Montería	40	0.15	0.53	0.32	0.41	0.59	-0.08
Los Córdobas	40	0.83	0.15	0.02	0.91	0.09	0.21
Montelíbano	40	0.85	0.15	0.00	0.93	0.07	-0.08
Lorica	40	0.65	0.30	0.05	0.80	0.20	0.06
Planeta Rica	40	0.92	0.08	0.00	0.96	0.04	-0.03
Pueblo Nuevo	40	0.65	0.35	0.00	0.82	0.18	-0.21

N: number of mosquitos evaluated; VV1016/FF1534/VV410: wild-type homozygotes (susceptible), VI1016/FC1534/VL410: heterozygotes, II1016/CC1534/LL410 mutant homozygotes (resistant). The inbreeding coefficient with a F_IS_ test.

Of a total of 560 females of *Ae*. *aegypti* evaluated, ten combinations of tri-locus haplotypes were identified. Of these, the wild homozygous triple haplotype (FF_1534_ VV_1016_ VV_410_) was found only in the Ayapel population, with a frequency of 0.05. On the other hand, the triple homozygous mutant haplotype (CC_1534_ II_1016_ LL_410_) was detected in the populations of Cereté, San Bernardo del Viento, Lorica, Valencia, Montería and Los Córdobas with frequencies between 0.02 and 0.32. The triple heterozygous haplotype (FC_1534_ VI_1016_ VL_410_) occurred only in Los Córdobas and Valencia populations with a frequency of 0.025.

**Fig 3:**
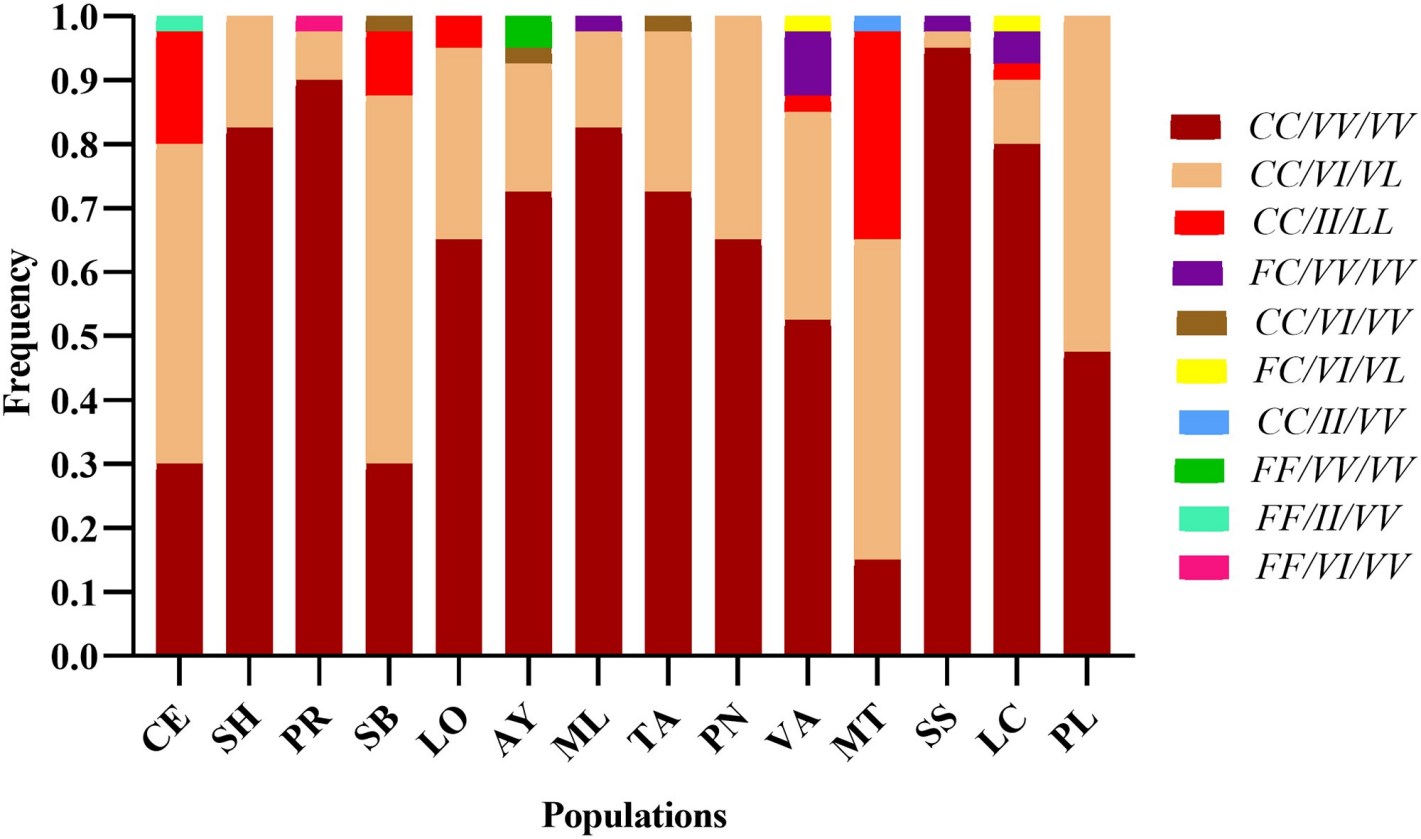
Frequencies of tri-locus haplotypes in each population of *Aedes aegypti* from Córdoba, Colombia. Haplotype order: 1534/1016/410. CE: Cereté, SH: Sahagún, PR: Planeta Rica, SB: San Bernardo del Viento, LO: Lorica, AY: Ayapel, ML: Montelíbano, TA: Tierralta, PN: Pueblo Nuevo, VA: Valencia, MT: Montería, SS: San Andrés de Sotavento, LC: Los Córdobas, PL: Puerto Libertador.

The homozygous resistant haplotype for locus 1534 and wild homozygous for loci 1016 and 410 (CC_1534_ VV_1016_/VV_410_), together with the homozygous recessive haplotype for locus 1534 and heterozygous for loci 1016 and 410 (CC_1535_ VI_1016_ VL_410_), were found to be the most predominant. These were observed in all the populations evaluated, showing frequencies between 0.15 and 0.95 for (CC_1534_ VV_1016_ /VV_410_) and between 0.25 and 0.57 for (CC_1535_ VI_1016_ VL_410_) (Fig 3).

### Association of haplotypes with resistance to pyrethroids by determining susceptibility with the CDC method (1)

OR analysis was performed to determine if there was an association between different haplotypes and resistance to permethrin and lambda-cyhalothrin, which was determined using the impregnated bottle test following CDC guidelines (S2 Table). This calculation was carried out by comparing the OR of each haplotype against the set of other haplotypes (Table 5).

**Table 5:** Association of haplotypes with resistance to the permethrin and lambda-cyhalothrin in *Aedes aegypti* from Córdoba, Colombia, evaluating susceptibility using the CDC method.

CDC Methodology			
Insecticide	Haplotypes	OR	Fisher *p-value*
**Permethrin**	CIL	6.37	4.07E-42
	CIV	0.612	2.27E-01
	CVL	0.271	3.90E-03
	CVV	0.224	1.52E-30
	FVV	0	1.00E+00
**Lambda-cyhalothrin**	CIL	1.565	0.848145
	CIV	Infinite	0.00039
	CVL	0	1.000.000
	CVV	0.3768	0.041183
	FVV	N/A	1.000.000

The CIL haplotype showed a significant positive association with permethrin resistance (OR = 6.370, *P* = 4.07e-42). On the contrary, the CIV haplotype showed a negative association with resistance to permethrin (0.612, P = 2.27e-01), suggesting that individuals with this haplotype were more susceptible to permethrin. Similarly, CVL (OR = 0.271, *P* = 3.90e-03) and CVV (OR = 0.224, *P* = 1.52e-30) haplotypes were not found to be associated with permethrin resistance. Finally, for the FVV haplotype, it was not possible to calculate OR because of the lack of individuals in one or both categories.

Regarding lambda-cyhalothrin resistance, the analysis revealed that the CIL haplotype was not significantly associated with resistance (OR = 1.1565, *P* = 0.84). The CIV haplotype was associated with resistance to lambda-cyhalothrin (infinite OR and *P* = 0.00039). Still, these results should be interpreted with caution because no dead individuals were recorded with this haplotype. On the other hand, the CVL haplotype could not be associated with resistance because no living individuals with this haplotype and only one dead individual were found. For the CVV haplotype, a significant association was found with susceptibility to lambda-cyhalothrin (OR = 0.3768, *P* = 0.041183). This suggests that individuals with the CVV haplotype are more likely to be susceptible. For FVV, this result was indeterminate because of the absence of data (0 alive and 0 dead for this haplotype).

Together, these analyses highlight the complexity of genetic interactions that influence insecticide resistance, underscoring the importance of considering genetic variability in the design of vector control strategies.

### Association of haplotypes with resistance to pyrethroid insecticides evaluating susceptibility with the WHO technique (2022)

Odds ratio (OR) analysis was carried out to explore the association between different haplotypes and resistance to permethrin, deltamethrin, and lambda-cyhalothrin determined through the WHO tube with insecticide-impregnated paper method. This calculation was carried out by comparing the OR of each haplotype against the set of other haplotypes (Table 6).

**Table 6:** Association of haplotypes with resistance to permethrins deltamethrin and lambda-cyhalothrin in *Aedes aegypti* from Córdoba, Colombia, evaluating susceptibility using the WHO method (2).

WHO Methodology			
Insecticide	Haplotypes	OR	Fisher *p-value*
**Permethrin**	CIL	3.19	0.000004
	CIV	0.66	0.387326
	CVL	0.48	0.001170
	CVV	0.2	0.009232
	FVV	NA	1.000.000
**Deltamethrin**	CIL	4.44	4.77e-34
	CIV	2.71	0.113
	CVL	2.92	0.014
	CVV	0.80	3.13E-39
	FVV	N/A	1.000.000
**Lambda-cyhalothrin**	CIL	3.93	0.000000
	CIV	8.95	0.013263
	CVL	1.05	1.000.000
	CVV	0.26	0.000000
	FVV	0.63	1.000.000

The analysis revealed significant variations in the association of haplotypes with permethrin resistance. The CIL haplotype showed a strong positive association with resistance (OR = 3.9, *P* = 0.000004), which implied a significant relationship with a high probability of survival post-treatment with permethrin. In contrast, CVL and CVV haplotypes showed a negative association with resistance (OR = 0.48, *P* = 0.001170 and OR = 0.20, *P* = 0.009232), respectively, suggesting that these haplotypes could be related to increased susceptibility to permethrin. The CIV haplotype did not show a statistically significant association (OR = 0.66, *P* = 0.387326), while for the FVV haplotype, it was not possible to calculate OR because of the lack of individuals in one or both categories. These findings indicate a clear relationship between certain haplotypes and the response to permethrin, which could have important implications for resistance management strategies in the population studied.

In regard to deltamethrin, the CIL haplotype showed a strong positive association with resistance, indicating a high tendency towards resistance in individuals carrying this haplotype (OR = 4.44, *P* = 4.77e-34). Although the CIV haplotype suggests a positive association with resistance, it was not found to be statistically significant. On the other hand, the CVL haplotype showed a positive and significant association with deltamethrin resistance (OR = 2.92, *P* = 0.014), and the CVV haplotype indicated a tendency towards susceptibility (OR = 0.80, *P* = 3.13e-39).

Regarding resistance to lambda-cyhalothrin, significant variations were observed in the association between haplotypes and resistance. Specifically, the CIV haplotype showed a strong association with resistance (OR = 8.95, *P* = 0.0133); this suggests that the presence of this haplotype is significantly related to survival to lambda-cyhalothrin. Likewise, the CIL haplotype was positively associated with resistance (OR = 3.93, *P*≤0.000000), indicating its possible role in resistance to lambda-cyhalothrin. The CVL and FVV haplotypes did not show a significant association with resistance, while the results for the CVV haplotype (OR = 0.26 and *P*≤0.00000) suggest that this haplotype is more related to susceptibility to lambda-cyhalothrin.

## Discussion

Córdoba is a department in Colombia that is considered endemic for dengue since it has eco-epidemiological conditions that facilitate the persistence of the transmission of this disease. This situation has generated intense selection pressure over time with pyrethroids on *Ae*. *aegypti* populations. However, most of the municipalities in the department lack baseline information on the susceptibility of vector populations to these insecticides. Only in the municipalities of San Bernardo del Viento, Pueblo Nuevo [28], and Montería [12, 17] are there previous studies in which resistance to some pyrethroids has been reported. The results of this work strengthen our knowledge about the state of susceptibility of the populations of this vector in this department by including populations of *Ae*. *aegypti* from other municipalities without a baseline of susceptibility to insecticides and by updating the status of the populations for which previous information was available in this area of the country.

In Colombia, both the WHO tube test and the CDC bottle test have been used to evaluate susceptibility to pyrethroids in adult *Ae*. *aegypti*. These tests have revealed variability in susceptibility to pyrethroids in most populations studied [12, 17, 28], which coincides with the results obtained in the present study. It is important to highlight the methodological differences between the two tests, considering that the WHO tube test measures the mortality rate of mosquitoes exposed (usually for 1 h) to a discriminating concentration of the insecticide for a specific time (regularly 24 h) [24] while the CDC bottle test determines the time necessary to incapacitate a susceptible mosquito using a predetermined concentration of insecticide [23]. It has been shown that the CDC method generates greater variability in mortality when compared to the WHO method, which may influence the actual interpretation of the susceptibility status of the populations evaluated [29]. The pyrethroids assessed by the CDC test may generate knockdowns in mosquitoes at the time of diagnosis, which does not necessarily generate mortality 24 h post-exposure [30]. Also, it has been shown that in the CDC bottle test, there may be poor accuracy in the results due to confounding variables such as deficiencies in bottle washing and volatility of the insecticide during the bottle impregnation and drying procedure that could also be influencing mortality as a variable of interest in the bioassay [31].

In this study for pyrethroids evaluated with the CDC methodology, mortalities between 15.8%–98.8% were observed for permethrin, between 98.5%–100% for deltamethrin and 90.3%–100% for lambda-cyhalothrin; through the WHO methodology, mortalities between 0%–19.4% were found for permethrin, 4.8%–88.5% for deltamethrin and 10.5%–85.2% for lambda-cyhalothrin. In a similar study, mortality rates between 30%–98% for permethrin, 99%–100% for deltamethrin, and 93%–100% for lambda-cyhalothrin were reported for populations in La Guajira [9]. Likewise, for other populations in the Colombian Caribbean, mortality percentages between 24%–77.6% for permethrin, 86%–100% deltamethrin and 43.3%–86.4% lambda-cyhalothrin were documented [17]. Also in populations in southwestern Colombia, mortalities between 94%–100% were found for deltamethrin and 81%–100% for lambda-cyhalothrin [32].

With respect to permethrin, resistance has been documented in populations of *Ae*. *aegypti* from the departments of Casanare, La Guajira, Atlántico, Cesar, and Córdoba [9, 12, 17, 33]. Specifically in the department of Córdoba, permethrin has not been used for vector control. However, in the present investigation, most of the populations evaluated showed a high intensity of resistance to this insecticide by the CDC bioassay and a high frequency of resistance with the WHO bioassay.

Studies carried out in the Caribbean region have shown susceptibility to deltamethrin through the bottle test for *Ae*. *aegypti* populations in the municipalities of San Bernardo del Viento, Pueblo Nuevo, and Montería in the department of Córdoba [12, 17, 28], as well as in the department of La Guajira [9]; the above coincides with the results obtained in the present study, taking into account that all the populations evaluated were found susceptible to deltamethrin with mortality between 98 and 100% using this methodology. Despite the above, there is evidence of resistance to deltamethrin in *Ae*. *aegypti* populations in other departments such as Bolívar, Cesar, Córdoba, Atlántico, Cundinamarca, Caquetá and Casanare [12, 22, 33].

In relation to lambda-cyhalothrin, some studies carried out in Colombia have recorded resistance to this pyrethroid using the CDC and WHO methods in populations of *Ae*. *aegypti* from the departments of Cundinamarca, Caquetá, Meta, Guaviare, Santander, Chocó, Antioquia, Putumayo, and Casanare [12, 22, 33–35]. Specifically, for the Colombian Caribbean region, different studies have been carried out in which the populations of Barranquilla, Puerto Colombia, Soledad, Valledupar, San Juan del Cesar, Sincelejo, Montería, Ciénaga, and Cartagena have been evaluated, finding resistance with the CDC bioassay [20]. Likewise, in a study carried out in this same region, the populations of Barranquilla and Juan de Acosta in the department of Atlántico, Chiriguaná in the department of Cesar, and Montería in the department of Córdoba were evaluated, where resistance was also found using the WHO test [17], and in a recent study carried out in the department of La Guajira, possible resistance in *Ae*. *aegypti* populations according to the CDC method was reported for the municipalities of Albania, Fonseca, Maicao, Riohacha, San Juan del Cesar and Villanueva with mortalities between 93 and 97% [9]. In the present work, susceptibility to lambda-cyhalothrin was recorded in twelve of the fourteen populations evaluated, and possible resistance in the populations of Montería and Cereté with respective mortality rates of 90 and 92%.

The resistance to pyrethroids observed in the department of Córdoba could be due to various causes, including cross-resistance with DDT [20]. Like pyrethroids, DDT similarly affects VGSC in insects [36]. Furthermore, the continued use of household insecticides by municipal residents could be generating selection pressure on populations of *Ae*. *aegypti* mosquitoes of the department.

Other possible causes of resistance to pyrethroids could be because Córdoba has agriculture, livestock, forestry, and fishing as its main economic activities [17], and it is likely that the constant use of pesticides could play a key role. It is also important to highlight that the department has malaria-endemic municipalities such as Montelíbano, Puerto Libertador, Valencia, and Tierralta, in which awnings impregnated with pyrethroid-type insecticides are used to control *Anopheles* spp., which can contribute to the development of resistance in *Ae*. *aegypti* populations [37].

Knockdown resistance (*kdr*) is a form of mosquito resistance to pyrethroid insecticides, primarily due to changes in one or more amino acids in VGSC. In different countries worldwide, *kdr* mutations have been identified in populations of *Ae*. *aegypti*: G923V, L982W, I1011M, V1016G, I1011V, S989P, D1763Y, T1520I, F1534G, A1007G V1016I, F1534C and V410L, with variations in their allelic and genotypic frequencies [25, 26, 34, 38–41]. In some countries in the Americas, the V1016I, F1534C and V410L mutations have been mainly associated with pyrethroid resistance in populations from Mexico [25, 42–46], Brazil [27, 47–51], Colombia [9, 12, 17, 20, 34], Peru [52], Costa Rica [53] Puerto Rico [54] Haiti [55], United States [56] and Venezuela [57, 58]. Specifically in Colombia, these mutations have been reported and associated with resistance to pyrethroids in populations of this vector in the departments of Antioquia, Valle del Cauca, Atlántico, Cesar, Bolívar, Magdalena, Sucre, Córdoba, La Guajira, Meta, Santander and Quindío [12, 17–20, 34, 59], with allele frequencies for V410L between 0.02 and 0.72, for F1534C between 0.44 and 1.0 and V1016I between 0.01 and 0.43 [9, 12, 17, 20, 34].

In the present work, it was identified for the first time in the populations of *Ae*. *aegypti* from the municipalities under study, the mutations F1534C with frequencies of the resistant allele C1534 between 0.94–1.0, V1016I with allele frequencies of I1016 between 0.01–0.43 and V410L with allele frequencies of L410 between 0.01–0.40; an exception was the population of Montería, which had two previous studies carried out in 2012 and 2018, respectively, and in which allele frequencies of 0.88 and 1.0 for the C1534 mutation, 0.33 and 0.70 for I1016 and 0.72 for L410 had been identified in the study carried out in 2018 since in 2012 there was no report of this mutation for the population of Montería [12, 17, 20]. The increase in the frequencies of the resistant alleles C1534 and I1016 was reported in these previous studies for *Ae*. *aegypti* from the municipality of Montería, may have been due to the selection pressure exerted with pyrethroids, especially lambda-cyhalothrin and deltamethrin, due to epidemic outbreaks of dengue and epidemics of chikungunya and Zika recorded in this period in Córdoba. Furthermore, in these previous studies, the C1534 allele showed an allelic frequency of 1, indicating that this mutation was fixed in this population, a confirmed finding in the present work. It is important to highlight that comparison of the allele frequencies reported by Pareja-Loaiza *et al*. (2020) for 2018 for I1016 (0.70) and L410 (0.72) with those observed in the present study (0.60 and 0.59, respectively) showed a decrease. This change could be related to the suspension of the use of lambda-cyhalothrin and deltamethrin, considering that since 2018 in Córdoba, these insecticides stopped being used to control dengue, replacing them with malathion.

Regarding the tri-locus haplotypes, ten combinations were identified in the analyzed populations, with the most frequent being CC/VV/VV and CC/VI/VL. The presence of these and the triple mutated haplotype CC/II/LL in the populations of Montería, Cereté, San Bernardo del Viento, Lorica, Valencia, and Los Córdobas probably conferring resistance to type I and II pyrethroid insecticides since the presence of the F1534C mutation alone confers resistance to type I pyrethroids such as permethrin [60].

In the present study, a positive association was found between the frequency of the tri-locus haplotypes CC/VV/VL, CC/VI/VL, and CC/II/LL with mortality for lambda-cyhalothrin, deltamethrin, and permethrin, respectively, suggesting that individually mutated alleles affect the observed resistance to these insecticides [61]. Considering the above, the presence of the F1534C mutation and the heterozygous and homozygous mutant genotypes V1016I and L410V present in the analyzed populations may be responsible for the resistance to pyrethroids found in the *Ae*. *aegypti* populations from the department of Córdoba.

Regarding the association of haplotypes with resistance to pyrethroids using the WHO test, the triple-resistant haplotype CIL showed a strong positive association with resistance to permethrin (OR = 3.19, *P* = 0.000004), lambda-cyhalothrin (OR = 3.39, *P* = 0.000000) and deltamethrin (OR = 4.44, *P* = 4.77e-34), the above coincides with a study carried out with populations of *Ae*. *aegypti* in Mexico, where the triple mutated haplotype was strongly associated with knockdown resistance and recovery for permethrin and deltamethrin [62]. In contrast, the CVL and CVV haplotypes showed a negative association with resistance (OR = 0.48, *P* = 0.001170) and (OR = 0.20, *P* = 0.009232), respectively, which follows that the haplotypes may be related to greater susceptibility to permethrin, differing from what was reported in a study carried out in populations from the Caribbean region where the CC/VI/VL haplotype was significantly associated with resistance to permethrin and lambda-cyhalothrin using the WHO test [17].

Regarding the results observed for the CIV haplotype, a strong association was found with resistance to lambda-cyhalothrin using the CDC-impregnated bottle test (OR = 8.95, *P* = 0.0133); these results coincide with a study reporting that co-occurrence of the F1534C + V1016I mutation enhances resistance to type I and II pyrethroids [60].

For the CVL haplotype, a significant positive association with deltamethrin resistance was observed (OR = 2.92, *P* = 0.014). These findings are consistent with a previous investigation [27], which indicates that the presence of the V410L mutation, alone or in combination with F1534C, confers high levels of pyrethroid resistance. Furthermore, the co-occurrence of the V1016I and V410L mutations with F1534C points towards two possible evolutionary models. The first suggests that the V1016I and V1410L mutations occurred independently in a haplotype that already contained C1534 and was subsequently associated with the latter by recombination in the cis configuration. The second model proposes that the three mutations arose independently and were organized in cis through two distinct recombination events [62].

According to the association of haplotypes with resistance to pyrethroids, in which the bioassays were carried out in CDC bottles, it was found that the CIL haplotype showed a significant positive association with resistance to permethrin (OR = 6.370, *P* = 4.07e-42). The above coincides with what was reported in Argentina, where the CIL haplotype was associated with resistance to permethrin but not to deltamethrin in bioassays carried out with the CDC method [63]. Additionally, in the present study, it was observed that the CVL (OR = 0.271, *P* = 3.90e-03) and CVV (OR = 0.224, *P* = 1.52e-30) haplotypes were not found to be associated with resistance to permethrin. These results are similar to those observed in populations from the Caribbean region where the CC/VI/VL or FC/VI/VL haplotypes showed a greater probability of being associated with susceptibility to permethrin and lambda-cyhalothrin evaluated using the CDC bottle test [17].

In light of these findings, a program of regular monitoring of the susceptibility of *Ae*. *aegypti* populations to pyrethroid insecticides is recommended in all municipalities of the department. It is important to consider the significant role that *kdr*-type mutations play in resistance to these insecticides. Specifically, the F1534C mutation, which occurs at high frequencies, together with both homozygous and heterozygous genotypes of V1016I and V410L, could confer adaptive advantages in the presence of pyrethroids, favoring a resistant phenotype in mosquito populations [62].

## Conclusions and recommendations

In this study, after evaluated the resistance to the pyrethroid insecticides evaluated can be attributed to the evolutionary advantage conferred on these populations of the *Aedes aegypti* mosquito by the presence of the *kdr* mutations F1534C, V1016I and V410L together. It is necessary to carry out constant monitoring in these populations to show changes in the frequencies of these mutations and additionally implement it in the remaining municipalities of the department where the current state of susceptibility to insecticides used in public health for vector control is not yet known.

## Supporting information

S1 Table OMS % mortality. (XLSX)

S2 Table *Kdr* phenotype. (XLSX)

S1 File Data analysis. (DOCX)
