# Supplementary material for: Assessing pyrethroid resistance in Aedes aegypti from Cordoba Colombia: Implications of kdr mutations
Source: PLoS One. 2024 Aug 22;19(8):e0309201. doi: 10.1371/journal.pone.0309201 (PMC11340990; doi:10.1371/journal.pone.0309201)
Supplement: S1 File — (DOCX) [file pone.0309201.s003.docx]

**Data analysis**

**WHO, CDC bioassay and resistance intensity**

The mortality of the tested sample is calculated by summing the number of dead mosquitoes in all exposed replicates and expressing it as a percentage of the total number of exposed mosquitoes:

$$observed mortality=\frac{total number of dead mosquitoes}{total number of mosquitoes exposed}\times100$$

A mortality between 98% and 100% indicates susceptibility in the mosquito population, mortality between 90 and 97% suggests possible resistance, and mortality less than 90% indicates resistance. Following this, the specimens are individualized according to their phenotype (alive and dead) for subsequent molecular analysis ( Kdr mutations ).

**Allele and genotypic frequencies**

From the parental F0 mosquitoes, the allele frequencies for I1016, C1534 and L410 are calculated and the following equation is used:

$$\frac{n heterozygotes+2 (n homozygous)}{2 (total n mosquitoes tested)}$$

The genotype frequencies for V _1016_ /V _1016_ , F _1534_ /F _1534_ , V _410_ /V _410_ , I _1016_ /I _1016_ , C _1534_ /C _1534_ , L _410_ /L _410_ , V _1016_ /I _1016_ , F _1534_ /C _1534_ , V _410_ /L _410_ are calculated as follows:

$$\frac{n mosquitoes with the genotype to calculate}{n total number of mosquitoes tested}$$

The inbreeding coefficient is additionally calculated using the following formula:

FIS = 1-( Hobs / Hexp )

### Where: Hobs is the number of observed heterozygotes and Hexp is the number of expected heterozygotes. If Fis is significantly greater than 0, it is considered an excess of homozygotes. If Fis is considerably less than 0, it is considered an excess of heterozygotes in the population, with a significance of (P< 0.05). Additionally, the frequencies of the tri-locus genotypes in the study populations are determined.

### **Association of *kdr* haplotypes with resistance to pyrethroids**

The genotypes observed in the exposed mosquitoes are grouped into haplotypes as shown in the following table:

Following this, the total number of living and dead belonging to each haplotype for each population is determined by insecticide, finally making the sum as shown in the following table:

Finally, to evaluate the association between the different haplotypes and resistance to insecticides, an OR analysis is performed.
